# Appropriate characterization of reservoir properties and investigation of their effect on microbial enhanced oil recovery through simulated laboratory studies

**DOI:** 10.1038/s41598-024-65728-4

**Published:** 2024-07-04

**Authors:** Neelam Kapse, Sumit S. Dagar, P. K. Dhakephalkar

**Affiliations:** 1 Bioenergy Group, MACS-Agharkar Research Institute, G.G. Agarkar Road, Pune, Maharashtra 411004 India; 2 Savitribai Phule Pune University, Ganeshkhind Road, Pune, Maharashtra 411007 India

**Keywords:** Biotechnology, Microbiology

## Abstract

Appropriate characterization of reservoir properties and investigation of the effect of these properties on microbial metabolism and oil recovery under simulated reservoir conditions can aid in development of a sustainable microbial enhanced oil recovery (MEOR) process. Our present study has unveiled the promising potential of the hyperthermophilic archaeon, identified as *Thermococcus petroboostus* sp. nov. 101C5, to positively influence the microenvironment within simulated oil reservoirs, by producing significant amounts of metabolites, such as biosurfactants, biopolymers, biomass, acids, solvents, gases. These MEOR desired metabolites were found to cause a series of desirable changes in the physicochemical properties of crude oil and reservoir rocks, thereby enhancing oil recovery. Furthermore, our study demonstrated that the microbial activity of 101C5 led to the mobilization of crude oil, consequently resulting in enhanced production rates and increased efficiency in simulated sand pack trials. 101C5 exhibited considerable potential as a versatile microorganism for MEOR applications across diverse reservoir conditions, mediating significant light as well as heavy oil recovery from Berea/carbonaceous nature of rock bearing intergranular/vugular/fracture porosity at extreme reservoir conditions characterized by high temperature (80–101 °C) and high pressure (700–1300 psi). Core flood study, which truly mimicked the reservoir conditions demonstrated 29.5% incremental oil recovery by 101C5 action from Berea sandstone at 900 psi and 96 °C, underscoring the potential of strain 101C5 for application in the depleted high temperature oil wells.

## Introduction

Petroleum oil reserves are finite and ever depleting. Overexploitation of this resource is as a primary factor for the declining crude oil production from existing reservoirs. Additionally, several factors including reservoir souring, reservoir heterogeneity, heaviness of residual oil, wellhead plugging, etc., collectively contribute to the significant decline in the oil production from the producer wells^1–4^. Such problems are complex and require coordinated efforts from diverse disciplines such as microbiology, geochemistry, and engineering.

The conventional oil recovery methods involving primary and secondary recovery often leave a significant amount of oil behind i.e., up to 60% of residual oil, trapped in the reservoir due to various factors such as capillary forces, rock permeability, etc^5^. Enhanced oil recovery (EOR) processes are often employed for unlocking the additional oil reserves. The drawbacks associated with EOR methods such as their high cost and associated difficulties with waste disposal have made the oil industry look for alternative cost-effective technologies such as microbial enhanced oil recovery (MEOR).

MEOR is an environmentally friendly and economically feasible process that utilizes microorganisms' potential to improve oil recovery from reservoirs. MEOR involves injecting indigenous or exogenous microorganisms and a suitable nutrient suite into the oil reservoir, to promote in situ microbial growth and desired metabolite production, contributing to enhanced oil production^6,7^.

Earlier, it was believed that harsh in situ conditions such as high temperature, pressure, and salinity make reservoir environment hostile to microbial growth^8,9^. Consequently, it was assumed that microorganisms played no role in such conditions. However, over the past few decades, it has been well established that petroleum reservoirs are not sterile environments but are conducive to microbial growth^10–12^ Microbial activities, thereby have been considered beneficial to petroleum exploitation^6,13^. Microorganisms are ubiquitous in subsurface environments of oil reservoirs and can significantly alter the properties of reservoir fluids and rocks, through the influence of their metabolites^14^. Understanding the impact of microbial metabolism and reservoir properties on EOR has emerged as a promising avenue to recoup this unproduced and unrecoverable oil.

Laboratory simulations face significant challenges in accurately replicating the complexity of oil reservoirs, which serve as intricate biological habitats^15–17^. While numerous studies on MEOR have been conducted in laboratories, the translation of these results to real-world scenarios becomes difficult due to the heterogeneity of reservoirs^18,19^. Although most MEOR experiments conducted at a smaller scale in the laboratory have shown promising outcomes^20,21^, it is challenging to predict the outcomes of applying MEOR techniques to a new oilfield based solely on the results of another oilfield study due to reservoir heterogeneity. A considerable number of trials have also been implemented in various parts of the world. The results of field implementation are mixed, some resulting in incremental oil production while some failing to do so^22–25^. The efficiency of MEOR in terms of oil recovery varied greatly between fields and between reservoirs, owing to different reservoir characteristics in different fields such as lithology, nature of the rock formation, porosity, permeability, temperature, crude oil properties. Also, the microbial formulation used, its concentration were the influencing factors in MEOR. Studies to explain the success or failure of MEOR field trials are severely limited by the insufficient consideration given to the reservoir conditions and their effect on microbial metabolism^23^.

The oil reservoir environment is extremely hostile, which can negatively impact the activity of externally introduced or stimulated microbes and consequently hinder the effectiveness of MEOR technique^26^. The harsh conditions within an oil reservoir, including extreme temperatures, pressures, salinity, and pH levels, directly influence microbial growth, metabolism and oil recovery efficiency^27^. Ghaffari et al.^28^, highlighted the significant influence of reservoir characteristics such as temperature and pressure on MEOR efficiency. Their study investigated the effects of pressure (500–2000 psi) and temperature (40–80 °C) on ex-situ MEOR using *Rhodococcus erythropolis*. Particularly, temperature of 40 °C and pressure of 2000 psi were found to positively influence oil recovery, resulting in a 20% increase based on original oil in place (OOIP)^28^. Previous studies of MEOR performed in simulated sand pack columns using microbial culture have reported oil recoveries ranging from 6 to 23.2%^29–33^. These studies had operational temperature in the range of 37–96 °C.

Another crucial factor is the reservoir's porosity, which affects the movement of microbes and determines their ability to grow and penetrate pores and crevices. Microbial processes can be compromised if the pore size is too small^34^. Hence, it's probable that the process of oil recovery through MEOR is largely influenced by pore morphology. Kogler et al.^29^, demonstrated the significance of porous media type on oil recovery efficiency. Their research investigated the effect of different porous media, namely glass beads, quartz sand and crushed reservoir rock samples on the oil recovery efficiency of *Halanaerobiales*. Sandpacks with pore diameters between 100 and 200 μm exhibited high permeability, facilitating the unrestricted transport of bacteria without plugging, consequently leading to incremental recovery^29^. The characteristics of the rock formation also indicate its susceptibility to weathering caused by microbial growth or acids, which can lead to the release of oil adhered to the reservoir rocks. However, laboratory studies on reservoir rocks are often limited due to the restricted availability of suitable analogs and the unsuitability of the rocks used^29^. Most MEOR studies, both in the laboratory and in the field, have focused on sandstone formations, with very few conducted on carbonate and fractured formations, which account for a significant portion of the world's crude oil storage^35^. This raises concerns about the applicability of MEOR in non-sandstone formations. Moreover, the properties of crude oil can significantly impact the implementation of the MEOR process, as highly viscous oil poses challenges to the extraction process^36^. Numerous studies have demonstrated enhanced oil recovery through the sand-pack columns or cores with different hydrocarbon mixtures. For instance, Gudina et al.^30^ assessed the impact of four distinct oil types namely, heating oil, viscous paraffin, Arabian Light crude oil, and heavy oil on the oil recovery potential of *Bacillus subtilis* strains. Their study revealed varying degrees of additional oil recoveries (AOR) depending on the hydrocarbon mixture and microorganism employed. The observed AOR was attributed to the biosurfactant-producing and oil-degrading capabilities of the microorganisms^30^. Also, externally introduced microbes would also have to compete with native reservoir species, which are better adapted to the environment. Additionally, low permeability of the rock formation, and high interfacial tensions between oil and water can present significant obstacles to efficient oil recovery through MEOR techniques^18^.

The success or failure of MEOR field trials often hinges on the lack of a comprehensive and quantitative understanding of microbial activity within the reservoir. To achieve optimal oil recovery, it is vital to identify the reservoir characteristics and operational parameters that influence microbial performance in MEOR^17^. This entails studying both reaction engineering and reservoir engineering aspects. Unfortunately, there is a significant dearth of mechanistic explanations for the increased oil recovery achieved through microbial processes in the reservoir, as well as a surprising lack of studies evaluating the effectiveness of microbial cultures in simulated reservoir conditions that consider all relevant parameters. Neglecting reservoir engineering perspectives severely limits the practical applicability and usefulness of simulated MEOR studies. Therefore, a thorough investigation and understanding of the impact of reservoir properties on MEOR effectiveness is essential.

Variations in reservoir conditions impact both the diversity as well as the efficacy of microbial enhanced oil recovery (MEOR). While numerous microbial cultures have been employed in MEOR applications, it's crucial to recognize that different reservoir types may require specialized microbial intervention. In our previous study, we documented the genetic and metabolic arsenal of hyperthermophilic archaeon, *Thermococcus petroboostus* sp. nov. 101C5 and showed its MEOR potential at 96 and 101 °C in sand pack trials^37^. In the present study, we report the impact of various reservoir conditions such as pressure, temperature, porosity, rock types, and crude oil types on the MEOR potential of strain 101C5. Further, to more accurately mimic the actual reservoir conditions, final trials were conducted in core flood apparatus using Berea Sandstone.

This manuscript presents a comprehensive analysis of the influence of microbial metabolism and reservoir properties on enhanced oil recovery, drawing upon insights obtained from simulated laboratory studies. By examining the results of these simulated laboratory studies, we aim to provide a deeper understanding of the intricate interactions between microbial metabolism and reservoir properties during enhanced oil recovery. Such insights hold significant potential for optimizing EOR strategies and developing sustainable approaches for oil production.

## Results and discussion

A hyperthermophilic anaerobic archaeon identified as a putative novel species, *Thermococcus petroboostus* sp. nov. was used in the present study. The strain 101C5 displayed potent MEOR desired property based on its ability to produce significant quantities of carbon dioxide (43.8 ml molasses g^−1^), acetate (1477 mg/l), lactate (466.4 mg/l) and emulsifying ability (EI24%: 60.8%) at 101 °C, as reported in our previous study^37^. These metabolites effected oil recovery at high temperatures in sand pack trials^37^. It was observed that biosurfactants emulsified the oil, acids reduced the viscosity, and CO_2_ pressurized the simulated reservoir environment. Even though, the recovery of residual oil was significantly enhanced using 101C5, there was scope for further improvement. Also, reservoir engineering analyses were essential for effective implementation of the MEOR process in field. Therefore, by accurately characterizing reservoir properties such as temperature, pressure, porosity, rock type (carbonaceous or otherwise), viscosity, and pour point of the oil, and studying their impact on microbial metabolism and MEOR under simulated reservoir conditions, a successful MEOR process can be developed for use in high-temperature depleted reservoirs.

Thus, the effect of following parameters on growth of microbial culture and microbe facilitated oil recovery was evaluated in present study.

### Temperature

**Figure 1:**
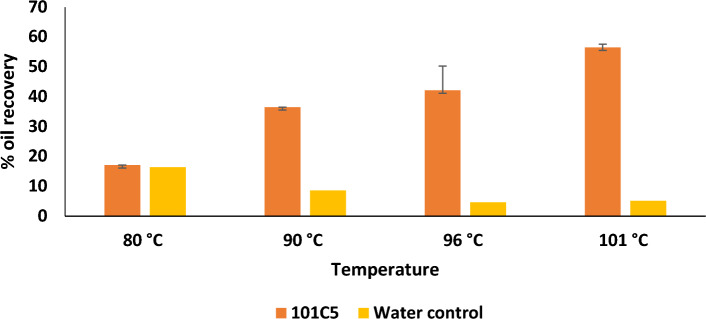
Oil recovery effected by 101C5 at different temperatures (*% Oil recovery potential of 101C5 at 96 °C and 101 °C^37^).

Microbes selected for MEOR applications must meet one critical prerequisite, which is, ideally, the ability to survive and produce the desired metabolic products in the reservoir. In most of the petroleum reservoirs, particularly in the Indian subcontinent, temperatures are expected to vary greatly but can be as high as 80 °C and may range to 100 °C. Thermophilic microbes possessing thermally stable enzymes are the most suitable candidates for MEOR application in such reservoirs as they have a stronger adaptive ability to survive extreme environmental conditions^38^. In the present study, the ability of 101C5 to grow, produce desired metabolites, and effect oil recovery at temperatures exceeding 80 °C was evaluated in crude oil-saturated sand pack column studies replicating reservoir environment in terms of temperature. It was observed that 101C5 could grow and produce metabolites which eventually effected oil recovery at all the temperatures tested. Additional oil recoveries of 17.1% and 36.5% were facilitated by 101C5 at 80 and 90 °C, respectively. The efficiency of oil recovered was far less in control columns where water was used instead of microbial culture under the same experimental conditions. 101C5’s oil recovery potential has also been documented at higher temperatures, 96 and 101 °C in our previously published manuscript^37^. 101C5 effected maximum oil recovery of 42.1% and 56.5% at 96 and 101 °C, respectively^37^. With increasing temperature, an increasing trendline in oil recovery was observed (Fig. 1). These results underscored the applicability of 101C5 for MEOR in high-temperature oil reservoirs.

It is important to highlight that the comparative assessment of MEOR efficiency of 101C5 could not be undertaken within the existing state-of-the-art framework due to the limited availability of information on MEOR across diverse reservoir conditions, especially at temperatures exceeding 90 °C.

### Pressure: 700–1300 psi

Hydrostatic pressure, the foremost physical characteristic of deep environments such as oil reservoirs, increases linearly with increasing depth. Inherently, strains indigenous to reservoirs would make the ideal candidates compared to other extremophilic microbes isolated from other environments^39^. Strains isolated from environments other than oil reservoir and are to be exogenously injected must grow and be metabolically active in regular reservoir conditions, having high pressure for MEOR to be successful. In the present study, 101C5 could grow and metabolize when exposed to a pressure range of 700–1300 psi, which was evident from the significant cell density as well as production of MEOR desired metabolites (Table 1). This observation, however, was not surprising as 101C5 was isolated from the reservoir itself. However, the ability of indigenous microbes to sustain the reservoir conditions needs to be validated, as behavior and activities of the microbes in a laboratory setup may not be a perfect representation of that in oil reservoir. With increasing pressure, a decrease in H_2_ production as well as in emulsification index was observed. 101C5 produced maximum metabolites in pressure range 900–1100 psi in 24 h. This study indicated the amenability of 101C5 for MEOR application in reservoirs having a pressure range of 700–1300 psi.

**Table 1:** Effect of pressure on growth and metabolite production by 101C5 desired for MEOR.

Pressure (psi)	Cell count (cells/ml)	H_2_%	CO_2_%	Acids (ppm)			Emulsification index (%)
				Succinate	Lactate	Acetate	
700	2.8 × 10^7^	6.8	16.8	124.5	134	106	38.6
900	3.6 × 10^7^	20.3	6.2	313.6	1008.3	224.7	38.6
1100	1.6 × 10^7^	9.8	6.4	435.5	2109.2	239.7	23.3
1300	1.2 × 10^7^	4.26	1.3	32	1593.6	90.3	21.1

Limited studies have documented the tolerance of microbes to extreme pressures since simulating reservoir pressure in laboratory is challenging.

### Porosity: intergranular/intercrystalline, vugular/solution, and fracture/matrix

Porosity and permeability are the most significant physical properties of the reservoir rock, which result from lithological, structural, and compositional behavior. Porosity is a measure of the capacity of reservoir rock to contain or store fluids. The porosity of the oil reservoir can significantly affect the displacement of oil and all EOR processes.

Numerous MEOR studies at laboratory level have assessed the effect of diverse groups of microorganisms and their metabolites on oil recovery; however, the effects of different porosity types on oil recovery have not been adequately documented, thus warranting further research in this area. Understanding how different porosities affect oil recovery will permit the prudent application of microorganisms for enhanced oil recovery.

**Figure 2:**
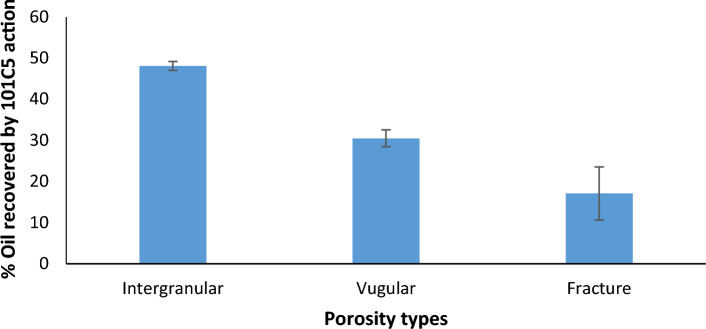
Oil recovery mediated by 101C5 in different porosity matrices.

Different types of porosity play a significant role in MEOR processes. This study evaluated the MEOR potential of 101C5 in three types of porosities: intergranular/intercrystalline, vugular/solution, and fracture/matrix porosity. For intergranular/intercrystalline porosity, the highly porous nature of beach sand (100 µm pore size) allowed unimpeded transport of 101C5, resulting in an additional oil recovery of 48.1%. In vugular/solution porosity, wherein the Naredi Limestone with cavities/ vugs (pore size: 0.1–5 µm) was used as a representative, incremental oil recovery of 30.5% was obtained. For fracture/matrix porosity, wherein sandstone-bearing fractures was used as a representative, 17.1% enhanced oil recovery was aided by 101C5. This study highlights that different types of porosities influence the activity of 101C5, and it proved to be a suitable candidate for MEOR in all three porosities, with better performance in intergranular porosity (Fig. 2). The selection of microbes for MEOR should consider their size relative to pore throats and ability to produce desired metabolites. 101C5, with a small cell size and desired metabolites, shows promise for successful MEOR applications in various oil reservoirs.

A majority of oil recovery studies have been carried out using sands^40–42^, while studies using carbonate and sandstone rocks with different porosity types are limited.

### Rock characteristics: *Berea* sandstone, carbonate rock

Oil reservoirs refer to accumulations of petroleum contained within porous or fractured rock formations, commonly found in sandstone, limestone, or shale sedimentary rocks. MEOR technology has proven to be highly effective in enhancing oil recovery from both sandstone and carbonate formations, as demonstrated by successful field trials^43^.

Owing to the limited availability of reservoir rocks, most MEOR studies have been conducted using outcrop sandstone or quartz sand to better simulate reservoir conditions^30,44^. To address the research gap and understand biogeochemical interactions between microbes and different lithologies, sand pack studies were conducted using crushed samples of reservoir sandstone and limestone. In this study, crushed reservoir rock samples with grain sizes < 1000 μm were used to pack metal columns (d = 3 cm, l = 15.5 cm, capacity = 60 ml). These columns were then inoculated with 101C5 and flooded with Molasses medium to investigate the following aspects: (i) influence of rock lithologies on microbial growth and metabolism, (ii) effect of microbial metabolites on rock geochemistry, and (iii) suitability of MEOR technology for incremental oil recovery.

Berea sandstone and Naredi Limestone were used in present study. 101C5 effected incremental oil recovery of 21.6% from Berea sandstone and 28.5% from crude oil-impregnated carbonate rocks as compared to water control, at 101 °C after 14 days shut-in period.

To gain a comprehensive understanding of the mechanisms underlying the oil recovery process, it is crucial to investigate the biochemical changes occurring in the system and their impact on both the rock and oil properties. Thus, in this study, we analyzed the metabolites produced by 101C5 within the sandstone and carbonate environments over a 14-day period. The production of various metabolites, including CO_2_, organic acids (acetic acid, formic acid, and lactic acid), EPS, and surface-active agents, was detected in the test column (Table 2); in contrast, none of these metabolites were detected in the control column. Biogenic production of significant quantities of CO_2_ was an important mechanism contributing to the oil recovery process by pressurizing the column and reducing the oil's viscosity, thus mobilizing the oil towards the surface. Notably, higher oil recovery was achieved in carbonate rocks, which could be attributed to the production of significant quantities of acetate, formate, and lactate (Table 2) compared to the sandstone experiment. Additionally, presence of succinate in carbonate sand pack, and its absence in sandstone sand pack, highlights the influence of rock lithologies on 101C5 metabolism. Furthermore, the emulsifying activity displayed by 101C5 in both rock types may have altered matrix wettability and reduced interfacial fluid tension, thereby enhancing oil recovery. A study conducted by Haddad et al.^45^, highlighted the significant role of surface active agents produced by *Bacillus persicus* in reducing the interfacial tension, resulting in the incremental oil recovery of 37.52% in carbonate cores. The EPS produced by 101C5 in simulated carbonate environment could have effectively plugged high permeability zones, diverting flow towards low permeability oil-rich zones, thus improving sweep efficiency and contributing to enhanced oil recovery. In summary, the metabolites produced by 101C5 interacted with oil as well as with the reservoir properties, resulting in crude oil mobilization, thereby demonstrating the significance of these biochemical processes in the oil recovery mechanism.

**Table 2:** Metabolite production by 101C5 after 14 days of incubation.

Rock type	CO_2_ (%)	E.I (%)	EPS (mg/ml)	Acids (ppm)			
				Acetate	Formate	Succinate	Lactate
Sandstone	15.6 ± 1.2	61.3 ± 6.5	0.75 ± 0.15	423.3 ± 8.0	156.2 ± 10.4	N.D	796.4
Carbonate	14.4 ± 1.5	55.8 ± 5.8	1.55 ± 0.05	797.2 ± 238.3	333.5 ± 139.3	202.4 ± 70.9	1534.2 ± 237.6

Based on these findings, 101C5 proved to be a promising candidate for MEOR in both sandstone as well carbonate nature of formations. Incremental recovery was highly dependent on the type of formation rock used, the highest oil recovery obtained in carbonate type. Rock properties such as permeability, pore size, and mineralogy play a crucial role in assessing the feasibility of MEOR because they directly influence the incremental recovery of oil. Hence, a comprehensive understanding of reservoir porosity, permeability, and mineralogy is essential for successful MEOR implementation, and these factors should be carefully considered when designing laboratory experiments.

Most of the research on MEOR has focused on sandstone formations compared to carbonate formations as carbonate types are geologically complex with substantial heterogeneity and, thus, challenging to manipulate and model in the laboratory. Very few reports on laboratory studies of MEOR in carbonate systems are available. For instance, sand pack studies with carbonate rocks demonstrated higher incremental oil recovery than pure quartz sand packs indicating wettability alteration, matrix dissolution, and plugging to be the primary mechanisms employed by *Halanaerobiales* for MEOR^29^.

### Oil properties: viscosity, gravity, pour-point

Gaining insight into the impact of the reservoir environment, including oil properties, is crucial to enhance the success of MEOR applications. Among various crude oil classes, heavy crude oil poses challenges in exploitation and recovery due to its high viscosity, density, and specific gravity compared to other oils^46,47^.

In this study, we examined four different types of crude oil with varying viscosity, gravity, and pour-point. These oils were collected from distinct oil wells in Ahmedabad, Gujarat, namely, Gandhar GGS-4, Kalol, Nandasan 34, and Shobhasan. The properties of each oil type are presented in Table S1.

Based on API gravity, the oil samples were categorized into light (> 31.1°), medium (31.1° to 22.3°), heavy (22.3° to 10°), and extra heavy (< 10°) crude oils (https://wiki.anton-paar.com/in-en/crude-oil/). Crude oil collected from Shobhasan and Gandhar oilfields was identified as light. Notably, Gandhar oil exhibited a high pour point, indicating its paraffinic nature compared to the other oils used. On the other hand, Kalol oil was classified as heavy, with an API gravity of 11.5° and a viscosity of 44.2 cP. Lastly, crude oil from the Nandasan oil field was characterized as extra heavy and highly viscous, possessing an exceptionally low API gravity of 8.04°.

**Figure 3:**
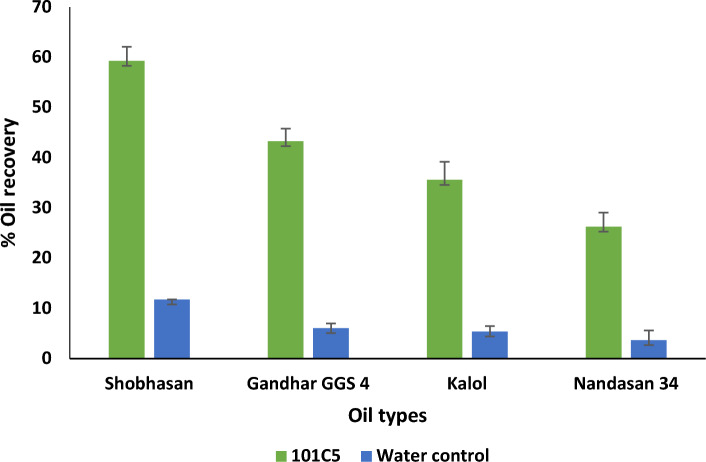
Oil recovery effected by 101C5 having different physical properties.

The ability of 101C5 to effect the recovery of different types of crude oil was evaluated in the sand pack column at 101 °C. The amount of oil displaced after primary recovery (water flooding) and the remaining oil in place after water flooding varied significantly depending on the oil type, likely influenced by differences in viscosities and API gravities of the oils. Additional oil recoveries ranging from 26.3 to 59.3% were observed with 101C5 in all cases of oil types tested as compared to water control (Fig. 3). 101C5 was most effective in recovering light oils from Shobhasan and Gandhar, facilitating maximum oil recovery of 59.3% and 43.3%. This ability may be attributed to the significant quantities of CO_2_ generated by 101C5, which may have acted on the paraffinic components of light oil, thereby degrading them as well as causing the pressurization of the sand pack column, pushing the oil towards the top, thereby facilitating oil recovery. Previous studies have demonstrated the role of biogenic gases like CO_2_ in the viscosity reduction of crude oil^30,48^.

101C5 also effected significant recovery of heavy oil from Kalol (35.6%) and Nandasan 34 (26.3%) compared to water control. The metabolites produced by 101C5, such as bio-emulsifier, might have emulsified the oil, while acetic acid might have reduced the viscosity of the heavy oil, allowing it to display improved flow features and thereby contributed to such enhanced oil recoveries. Organic acids are known to play a crucial role in the viscosity reduction of heavy crudes, thereby improving their flow characteristics. Laboratory simulated experiments based on the physico-chemical conditions of Daqing Oilfield demonstrated the role of acids in viscosity reduction from 28.1 to 18.0 6mPa s^49^.

Overall, these findings suggest that 101C5 has potential as a MEOR candidate in a wide range of oil reservoirs, offering promising prospects for improving oil recovery in diverse oil types.

### Effect of microbial performance on enhanced oil recovery using core flood study

The oil recovery potential of 101C5 was evaluated through a core flooding experiment designed to mimic actual reservoir conditions. To simulate the reservoir rock characteristics, a Berea sandstone core was selected. The core flood study were conducted at 96 °C and 900 psi over a period of 14 days, using light paraffinic oil (42° API). The core was saturated with the crude oil and microbial culture 101C5 along with Molasses medium, and after the incubation period, brine flooding was performed to determine the percentage of additional oil recovery.

The percentage of additional oil recovery mediated by 101C5 was calculated as follows:
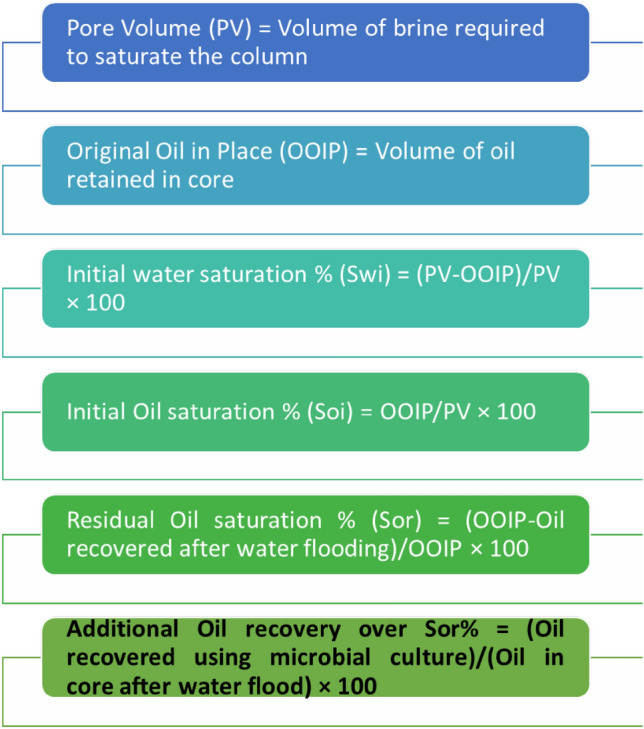


#### Oil and sandstone core characterization

The crude oil used in the present study was characterized as light paraffinic with an API gravity of 42° and a high pour point of 36 °C. The Berea sandstone characteristics and other oil recovery conditions are described in Table 3.

**Table 3:** Petro physical properties of core-flood assay.

Characteristics/conditions	Value
Type of rock sandstone	Berea
Length (cm) Diameter (cm) Pore Volume (ml)	6.9 2.4 31.1
Crude oil	Gadhar GGS-4
Nature of oil API gravity Pour point	Light paraffinic 42° 36 °C
Operation conditions	
Microbial culture Inoculum concentration (%) Brine, NaCl (%) Temperature (°C) Pressure (Psi) Incubation period	101C5 5 2 96 900 14 days

#### Oil recovery

During the core flood study, incremental oil recovery was observed at each stage. Initially, 1.8 ml of oil was recovered during the waterflooding stage, leaving 14.2 ml of Original Oil in Place (OOIP) in the Berea core. The injection of brine continued until no additional oil was released. After the waterflooding stage, 88.75% of the residual oil remained in the core. Next, 1200 ml of Molasses medium, inoculated with 5% of the microbial culture 101C5, was passed through the core, initiating MEOR process. On Day 0, during the lag phase of growth, the oil recovery was 1.2 ml, indicating negligible biomass and metabolite production. The core was kept under these conditions for 14 days. After the confinement period, a recovery stage was carried out with brine injection, resulting in an additional oil recovery of 3.0 ml. In total, 42.2% of the initial oil was recovered through waterflooding and MEOR, with 29.5% attributed to the activity of the 101C5 (Table 4). The presence of pore structure heterogeneities makes the Residual Oil (RO) remaining after waterflooding particularly challenging to mobilize with tertiary oil recovery methods, including MEOR.

**Table 4:** Cumulative light oil recovery facilitated by 101C5.

Parameters	Value
Initial oil saturation, S_Oi_ (%)	51.4
Initial water saturation, S_wi_ (%)	48.5
OOIP original oil in place (ml)	16
Residual oil saturation (Sor) %	88.75
Additional % oil recovery facilitated by 101C5	29.5

Compared to other studies, the oil recovery achieved in this study was significantly higher. 12.09% additional oil recovery was reported using a polymer-producing strain of *Enterobacter* in core flood study at 30 °C^50^. Sharma et al.^51^, reported 19% additional oil recovery by thermophilic *Clostridium* sp. at 65 ˚C. Another study using thermophilic *Clostridium* sp. reported a 10.1% oil recovery at 96 °C^32^.The remarkably high oil recovery in the present study is attributed to the metabolites produced by the strain 101C5, which likely altered the properties of the oil and core, facilitating the displacement of the residual oil. This study stands out as the first to report such a high oil recovery in simulated lab studies at 96 °C. Additionally, further analysis was conducted to identify the specific metabolites contributing to the oil recovery by 101C5.

#### Metabolite production

Actively growing cells of 101C5 were detected in the spent medium after 14 days, indicating the ability of 101C5 to survive and proliferate in the simulated environment mimicking the harsh oilfield conditions. The production of desired metabolites such as organic acids (acetic acid, lactate, and succinate), EPS, and surface-active agent, was detected from the spent medium collected after 14 days from the sealed core; however, none of these metabolites were seen at the waterflooding stage. In the spent medium, EPS was produced at a concentration of 3.2 mg/ml. Several laboratory and field studies have demonstrated the potential of polymer flooding and reported to increase oil recovery by displacement of entrapped oil by improving sweep efficiency by selectively plugging the high permeability zones within the rocks^52–54^. On such study exhibited oil displacement of 25.7% in sand pack studies due to the action of Biopolymer produced by *Rhizobium viscosum*^55^. Acetate, succinate, and Lactate at concentrations of 362.9 ppm, 71.2 ppm and 2319.4 ppm, respectively, were the primary metabolites produced by 101C5 in the confinement period of 14 days (Table 5). Significantly high acids produced by 101C5 contributed to oil recovery by modifying the oil and rock properties. Different studies have highlighted the importance of organic acids in the improvement of oil recovery. Ohno et al.^56^, reported acetic (280 ppm), butanoic, and propionic acids in a MEOR field trial to be the main contributors to increased oil production from the reservoir. Jinfeng et al.^57^, reported oil recovery of 5–10% in core flood study with acetic acid (135–217 ppm) to be the main metabolite.

**Table 5:** Concentration profile of the 101C5 produced metabolites in the oil recovery process.

Day	0	14th
Cell count (cells/ml)	5.5 × 10^6^	4.0 × 10^7^
pH	7.0	4.2
E.I (%)	0	49
EPS (mg/ml)	0	3.2
Acetate (ppm)	0	362.9
Succinate (ppm)	0	71.2
Lactate (ppm)	0	2319.4

Spent medium harboring 101C5 displayed an emulsifying activity of 49%, providing evidence for 101C5’s ability to produce surface-active agents in simulated reservoir conditions. These surface-active agents emulsify the crude oil, thereby enhancing oil mobility. Biosurfactants are the preferred agents for oil recovery operations. Haloi et al.^58^, reported a 16.7% increase in oil recovery by the action of biosurfactants produced by *Pseudomonas* sp. TMB2. The biosurfactant synthesized by *Clostridium* sp. mobilized 17.15% of residual oil in sand pack studies^59^. Fulazzaky et al.^60^, reported a biosurfactant producing thermophilic *Geobacillus* sp. to mobilize > 14% of residual oil in core flood studies at 60–70 °C.

In the present study, the surface-active agents, organic acids, and EPS produced by 101C5 were the major metabolites contributing to oil recovery. The mechanisms involved in incremental oil recovery were the emulsification of crude oil by surface-active agents and changes in the physicochemical properties of oil and the reservoir rock by the action of EPS and organic acids. The higher yield obtained by 101C5 with light crude underscored its potential for MEOR applications in high-temperature light oil reservoirs.

Previous studies of MEOR have reported oil recovery yields ranging from 7.7 to 34.3% in simulated core flood studies using Berea sandstone^50,51,58,61–63^. However, most of these studies were limited to temperature ≤ 70 °C. Only one report has documented the application of MEOR at temperatures 90 °C or above^32^. Our investigation achieved a comparatively higher oil recovery rate of 29.5% in core flood studies at 96 °C, employing the hyperthermophilic *T. petroboostus* sp. nov.

## Conclusion

The current investigation provides a comprehensive analysis of how various reservoir properties, including pressure, porosity, rock types, and oil types along with temperature, influence the oil recovery potential of hyperthermophilic archaeon *Thermococcus petroboostus* sp. nov. 101C5. Our study revealed noteworthy insights regarding the intricate relationship between reservoir characteristics and the efficiency of MEOR. Within a pressure range of 700–1300 psi, strain 101C5 demonstrated robust growth and metabolite production, with optimal outcomes observed between 900 and 1100 psi. Additionally, a positive correlation between temperature and oil recovery potential of 101C5 was noted. Strain 101C5 facilitated substantial additional oil recovery across different porosity types (intergranular, vugular and fracture), with its superior performance observed in intergranular porosity. Evaluation of MEOR efficiency in diverse rock lithologies (Berea Sandstone, Carbonate Rock) revealed substantial incremental oil recovery, particularly in carbonate formations. The metabolic activities of 101C5 significantly contributed to enhanced oil recovery across varying reservoir conditions. Also, significant incremental oil recovery was demonstrated by 101C5 across various crude oil types of varying viscosity, gravity, pour-point, with particularly high recovery observed with light crude oil. These results highlighted the immense potential of 101C5 to recover residual oil from a wide range of reservoirs having either Berea or carbonaceous nature of rock with intergranular, vugular or fractured porosity bearing 700–1300 psi pressure and 80–101 °C temperature and having different types of oil (heavy/light). Furthermore, 101C5 presented the ability to recover light crude oil from Berea sandstone in core flood trial, effecting > 29% recovery in 14 days, which is the highest oil recovery reported till date by any bacteria/archaea. In summary, our findings underscore the adaptability and efficacy of strain 101C5 for MEOR applications across a spectrum of reservoir parameters. The findings and insights presented herein contribute to the ongoing efforts to harness the potential of microbial processes and optimize EOR techniques for efficient and sustainable oil production.

## Materials and methods

### Bacterial culture and growth conditions

An archaeal strain 101C5 was isolated from produced water sample collected from Nandasan 9, an oil reservoir situated in Ahmedabad (Gujarat), India, and previously characterized by Kapse et al.^37^. Strain 101C5 was identified as a putative novel member of the genus *Thermococcus* based on ANI and DDH analysis, for which the name *Thermococcus petroboostus* sp. nov. was proposed. The strain 101C5 was grown in Molasses medium prepared in serum bottle (molasses 7.0%, yeast extract 0.5%, NaCl 5% supplemented with 4% SS-30 trace element solution (K_2_HPO_4_ 0.1%, KH_2_PO_4_ 0.1%, NaHCO_3_ 1%, CaCl_2_ anhydrous 0.02%, NaCl 0.2%, MgSO_4_ 0.02%) at 101 °C under anoxic conditions.

### Hydrocarbon mixture

Crude oil having an API gravity of 19°, collected from Viraj oilfield located in Ahmedabad region, Gujarat was used for simulation studies.

### Effect of reservoir properties on MEOR

#### Temperature

Sand pack experiments were conducted at temperatures exceeding 80 °C in 60 ml metal columns to investigate the effect of temperature on microbial enhanced oil recovery (MEOR). Columns were packed with 30 ml of acid-washed fine sand saturated with crude oil. After primary recovery using water, each column was inoculated with 101C5 and Molasses-based medium and incubated for 14 days at each temperature. In the control column, water was used instead of inoculum. Sand pack trials were performed in triplicates. After 14 days of incubation at respective temperatures, oil recovery was assessed, and % oil recovery was calculated as (Oil recovered after 14 days/Oil in place)*100. (*Oil in place = Void volume−Oil recovered after primary recovery).

### Pressure

The influence of pressure on the growth of strain 101C5 and metabolite production was assessed using a sealed core flood apparatus. Experiments were conducted at 96 °C and under pressure conditions of 700, 900, 1100, and 1300 psi for 24 h. After incubation, the spent medium was collected in sealed N_2_-flushed 1 L bottle, and metabolite analysis was performed.

#### Porosity: intergranular/ intercrystalline, vugular/solution, and fracture/matrix

##### Intergranular/intercrystalline porosity

Fine sand (0.1–0.25 mm) was used as a representative of Intergranular/intercrystalline porosity. Crude oil saturated sand packed columns were subjected to the same experimental procedures as described above for sand pack studies. After 14 days of incubation at 101 °C, oil recovery was assessed.

##### Vugular/solution porosity

Naredi Limestone from the Kutch basin, featuring vugs (cavities), was used as a representative of vugular porosity. Crude oil-saturated limestones were weighed to determine the void volume. Primary recovery of oil from stones was performed using water, and the amount of oil displaced was measured. The stones were then inoculated with 101C5 of cell density 10^7^ cells/ml with molasses-based growth medium. The test and control limestones (inoculated with water) were incubated at 101 °C for a period of 14 days, after which the oil recovery effected by culture and water was measured.

##### Fracture/matrix porosity

Sandstone was used to simulate fracture porosity in this study, which was saturated with crude oil. The amount of primary oil recovery, as well as residual oil in place, was determined. The test sandstones were subsequently saturated with molasses medium, inoculated with the hyperthermophilic culture 101C5 of cell density 10^7^ cells/ml, while the control was flooded with water and incubated at 101 °C for 14 days. The efficiency of the MEOR was calculated by subtracting the residual oil remaining in place after MEOR from the residual oil in place after primary recovery.

### Rock characteristics: *Berea* sandstone, carbonate rock

Owing to limited availability of core samples, reservoir rock samples (Sandstone and Carbonate) were crushed, and only grains < 1000 μm were used as packing material in metal columns (d = 3 cm, l = 15.5 cm, capacity = 60 ml). All materials were heat-sterilized (100 °C overnight) prior to use. To establish anaerobic conditions, the columns were flushed with Nitrogen gas and saturated with Brine followed by crude oil. The packed columns were then subjected to primary recovery and subsequently inoculated with the hyperthermophilic culture 101C5 and Molasses medium and incubated at 101 °C for 14 days, after which the oil recovery was made. Water was used instead of 101C5 in the control column.

### Oil properties: viscosity, gravity, pour-point

Four different oils of varying viscosity, gravity, and pour-point were used in the study. These oils were collected from different oilfields of Ahmedabad, Gujarat, namely, Gandhar GGS-4, Kalol, Nandasan 34, and Shobhasan. The ability of 101C5 to enhance recovery of four different types of oil was evaluated in sand pack experiments at 101 °C and MEOR was determined after 14 days. The sand pack experiment was performed as described earlier. All the experiments were performed in triplicate. Water was used instead of the microbial culture in the control column.

### Effect of microbial performance on enhanced oil recovery using core flood studies

A core flooding experiment was carried out in the pressure tapped core holder. The experimental system consisted of a continuous Upstream/ injection pump, an oven for temperature control, gas cylinders, a pressure meter, a differential pressure transducer, and a stainless steel core holder. Gas cylinders were used to displace fluids (brine, oil, and microbe plus nutrients). A differential transducer was fixed to monitor changes in pressure. Upstream/Injection pressure, overburden pressure, differential pressure (dPi) and backpressure were monitored throughout the experiment. A collector was used to collect the effluent of the experiments. Prior to setting-up of the core-flooding study, internal tubing of the apparatus was flushed with nitrogen gas (99.9% purity) to get rid of atmospheric oxygen. Nitrogen gas was used to maintain the desired pressure and anaerobic condition in the core-flooding apparatus throughout the experiment.

Briefly, the core was cleaned from hydrocarbons using benzene and methanol in the proportion of 75:25 vol/vol. After cleaning, the core was dried at 100 °C for 24 h. Berea sandstone of 6.9 cm length and 3.2 cm diameter was tightly packed in Viton sleeve and placed in the core holder assembly made up of stainless steel. The core was saturated with formation brine of 2% salinity using vacuum desiccators for 24 h, and pore volume (PV) was determined using the dry and wet weights of each core. The core was then flooded with crude oil (Light oil from Gandhar) until no more water was removed in order to create irreducible water saturation (S_wi_). The core was subjected to water flooding until no further oil was removed. The residual oil was calculated by measuring the amount of oil recovered by water-flooding. The oil saturation under this condition was considered to be original oil in place (OOIP). The core was then flooded with brine (1st brine flooding) until irreducible oil saturation (S_or_), where no more oil was produced. After preliminary operations, the model was incubated for 24 h at the experimental temperature and pressure values. Then, 5% of the hyperthermophilic culture 101C5 and the Molasses medium (650 ml) were injected as a tertiary recovery stage, and extra oil recovery was determined. The core flood study was conducted at 900 psi and 96 °C to mimic the pressure and temperature of the oilfield, respectively. The sealed core was incubated for 14 days. Subsequently, oil was recovered by displacement at the same temperature with brine. Aqueous (spent medium) and oil phases were obtained in the recovery stage, which were separated for subsequent analysis. % oil recovery was calculated as amount of oil recovered/amount of oil present in the core after primary recovery with water *100. In the aqueous phase, metabolites and biomass were determined. The recovered oil was quantified and analyzed.

### Supplementary Information

Supplementary Information.

## Data Availability

Data generated during the study is provided within the manuscript and supplementary information file.
