# Supplementary material for: Appropriate characterization of reservoir properties and investigation of their effect on microbial enhanced oil recovery through simulated laboratory studies
Source: Sci Rep. 2024 Jul 4;14:15401. doi: 10.1038/s41598-024-65728-4 (PMC11224412; doi:10.1038/s41598-024-65728-4)
Supplement: Supplementary file 1 — Supplementary Information. [file 41598_2024_65728_MOESM1_ESM.pdf]

Table S1. Physical properties of the Crude oil: viscosity (cP), API gravity (API) and pour point

Crude oil	Viscosity (cP)	API gravity (˚)	Pour point (˚C)	Nature of oil
Gandhar GGS-4	19.1	42	36 °C	Light paraffinic
Kalol	44.2	11.5	24 °C	Heavy
Shobhasan	13-24	33	33 °C	Light
Nandasan 34	--	8.04	27 °C	Extra heavy
